# Transcriptomic data reveals the dynamics of terpenoids biosynthetic pathway of fenugreek

**DOI:** 10.1186/s12864-024-10253-x

**Published:** 2024-04-22

**Authors:** Sara Lamei Javan, Arman Beyraghdar Kashkooli, AbdolAli Shojaeiyan, Sina Majidian

**Affiliations:** 1 Department of Horticultural Science, Faculty of Agriculture, Tarbiat Modares University, Tehran, Iran; 2 Department of Computational Biology, University of Lausanne, Lausanne, Switzerland; 3 Switzerland SIB Swiss Institute of Bioinformatics, Lausanne, Switzerland

**Keywords:** Elicitor, Methyl jasmonate, RNA-sequencing, Transcriptomic analysis, *Trigonella foenum-graecum*

## Abstract

Medicinal plants are rich sources for treating various diseases due their bioactive secondary metabolites. Fenugreek *(Trigonella foenum-graecum*) is one of the medicinal plants traditionally used in human nutrition and medicine which contains an active substance, called diosgenin, with anticancer properties. Biosynthesis of this important anticancer compound in fenugreek can be enhanced using eliciting agents which involves in manipulation of metabolite and biochemical pathways stimulating defense responses. Methyl jasmonate elicitor was used to increase diosgenin biosynthesis in fenugreek plants. However, the molecular mechanism and gene expression profiles underlying diosgening accumulation remain unexplored. In the current study we performed an extensive analysis of publicly available RNA-sequencing datasets to elucidate the biosynthesis and expression profile of fenugreek plants treated with methyl jasmonate. For this purpose, seven read datasets of methyl jasmonate treated plants were obtained that were covering several post-treatment time points (6–120 h). Transcriptomics analysis revealed upregulation of several key genes involved in diosgenein biosynthetic pathway including Squalene synthase (SQS) as the first committed step in diosgenin biosynthesis as well as Squalene Epoxidase (SEP) and Cycloartenol Synthase (CAS) upon methyl jasmonate application. Bioinformatics analysis, including gene ontology enrichment and pathway analysis, further supported the involvement of these genes in diosgenin biosynthesis. The bioinformatics analysis led to a comprehensive validation, with expression profiling across three different fenugreek populations treated with the same methyl jasmonate application. Initially, key genes like SQS, SEP, and CAS showed upregulation, followed by later upregulation of Δ24, suggesting dynamic pathway regulation. Real-time PCR confirmed consistent upregulation of SQS and SEP, peaking at 72 h. Additionally, candidate genes Δ24 and SMT1 highlighted roles in directing metabolic flux towards diosgenin biosynthesis. This integrated approach validates the bioinformatics findings and elucidates fenugreek’s molecular response to methyl jasmonate elicitation, offering insights for enhancing diosgenin yield. The assembled transcripts and gene expression profiles are deposited in the Zenodo open repository at 10.5281/zenodo.8155183.

**Supplementary Information:**

The online version contains supplementary material available at 10.1186/s12864-024-10253-x.

## Introduction

Fenugreek (*Trigonella foenum-graecum*) is one of India’s oldest medicinal plants from the Fabaceae family and is generally used in Ayurvedic medicine. This versatile plant serves both edible and non-edible purposes, encompassing applications in spices, fodder, dietary supplements, pharmaceuticals, and therapeutic practices [1].

Plants produce a large and diverse group of organic compounds called secondary metabolites, which can have medicinal properties. Thus, these metabolites have a very high economic value, although, they have a complex structure and their chemical synthesis is usually complex and expensive, hindering their development and investigation. Nonetheless, given their significant value, particularly in medicinal industries, understanding their chemistry, activity, and biosynthesis becomes imperative [2].

Fenugreek is a source of a biogenic steroid which is important in the pharmaceutical industry. It can be a suitable alternative to sweet potatoes, due to their high content of a steroid saponin called *diosgenin* [3]. Diosgenin has many applications in the pharmaceutical industry. For example, diosgenin is used for the production of steroid-type drugs. Despite their immense importance, products derived from secondary metabolites are naturally produced in limited quantities. For this reason, researchers seek methods to increase the production of these compounds in plants.

The production of secondary metabolites in plants can be stimulated through applying elicitors [4]. Elicitors are (a)biotic factors that activate the defense response of the plants against e.g., herbivores. One of the well-known elicitors is Methyl jasmonate, which is an important organic compound that plays a variety of regulatory roles in plant growth and development including axis elongation during embryogenesis, flower development, leaf senescence, root formation, and stomatal opening [5, 6]. It has been shown that treatment with 0.01% methyl jasmonate increased diosgenin levels by 10.5 times in fenugreek seedlings [7]. In addition, methyl jasmonate upregulated the expression level of two pivotal genes of the mevalonate pathway, the metabolic route leading to diosgenin: 3-hydroxy-3-methylglutaryl-CoA reductase and sterol-3-β-glucosyl transferase [7–8]. The application of exogenous jasmonic acid and methyl jasmonate is responsible for the induction of reactive oxygen species and subsequent defense mechanisms in cultured cells and organs [9]. It is also responsible for the induction of signal transduction, the expression of many defense genes followed by the accumulation of secondary metabolites. In other words, the accumulation of secondary metabolites in plant, cell, and organ cultures often occurs when cultures are subjected to varied kinds of stresses including elicitors or signal molecules.

There are many research studies focused on the effect of elicitors on secondary metabolites biosynthesis in medicinal plants, however, the underlying molecular mechanisms involved are not well studied. To understand the role of biosynthetic pathway genes and the effect of elicitors in increased biosynthesis and accumulation of diosgenin and other secondary metabolites, the expression patterns of genes involved in the biosynthesis pathway should be investigated.

**Fig. 1:**
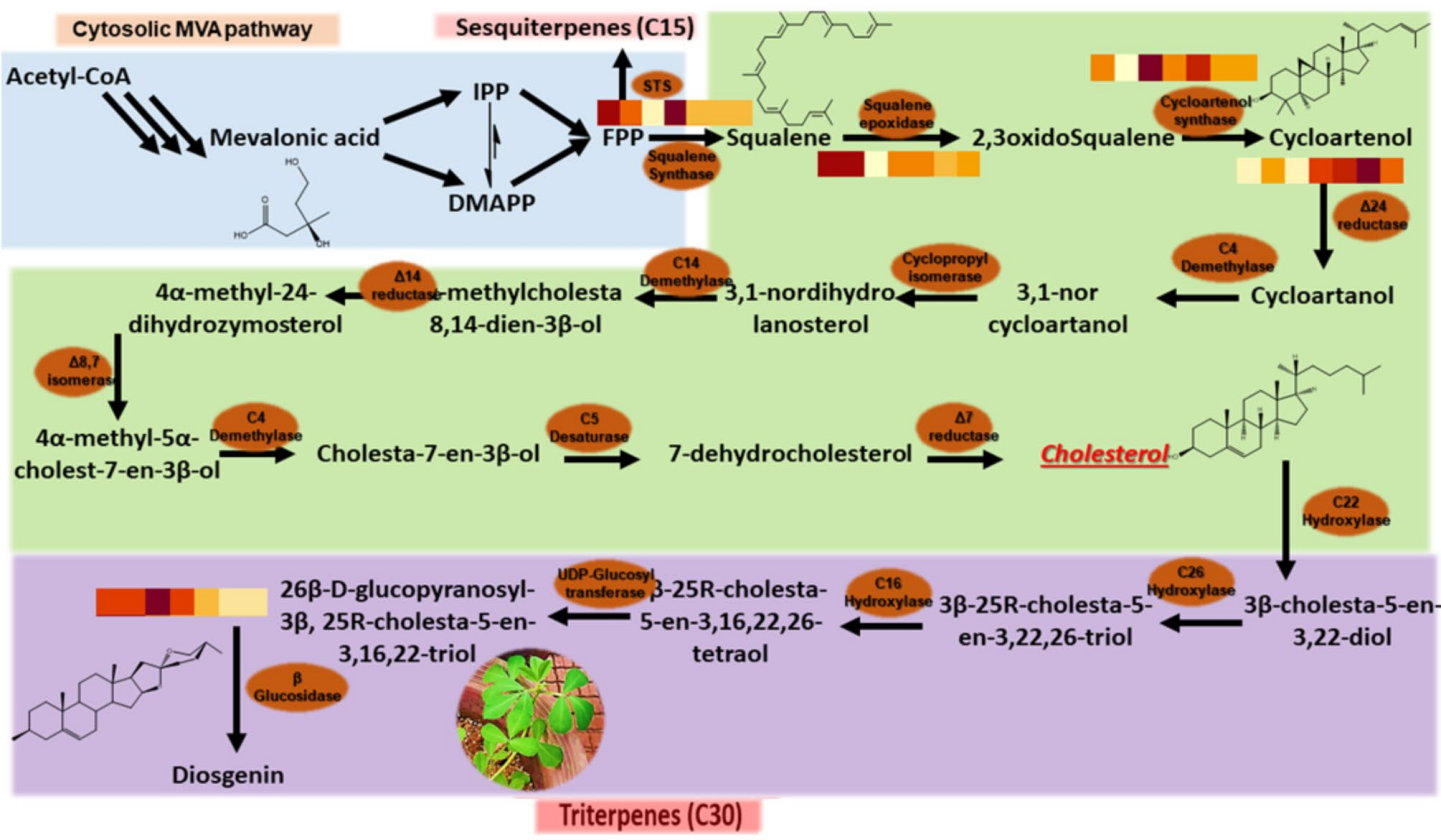
Biosynthetic pathway of diosgenin in fenugreek. Acetyl-coenzyme A (acetyl‐CoA) converts to cycloartenol via a series of multiple reactions. Cycloartenol is converted to either cycloartenol, leading to cholesterol formation (blue box), or 24 methylene cycloartenol, resulting in stigmasterol (green box). Cholesterol is then converted to 3β‐cholesta‐5‐en‐3,22‐diol, which ultimately results in the diosgenin biosynthesis (purple box). (Adapted from [10]). The heatmap shows the impact of treatment on expression changes of six genes on the biosynthetic pathway of diosgenin in fenugreek (see Result section for details)

To engineer the biosynthetic pathway, it is necessary to identify the biosynthetic pathway genes. Despite the fact the diosgenin biosynthetic pathway is partially identified (Fig. 1) [10], the data required for analyzing the molecular behavior of diosgenin biosynthetic pathway genes in fenugreek (e.g., RNA-sequencing or microarray analysis) is limited especially in response to elicitors [11]. It has been shown that meta-analysis can provide candidate genes for future research to improve our understanding of functions of secondary metabolites [12].

The RNA-seq approach has been extensively used in the literature to characterize the expression profile of different samples and find differentially expressed genes (DEGs) [6, 13, 14]. To better understand the effect of methyl jasmonate on secondary metabolites of fenugreek and molecular mechanisms in secondary metabolite accumulation, in this study, we benefit from the raw RNA sequencing reads and performed *de novo* assembly to infer the transcriptome. DEGs during six different treatment stages of fenugreek compared to the control sample were found. Besides, an extensive Gene Ontology (GO) enrichment was conducted to identify important biological processes and molecular functions involved.

## Results

### De novo assembly of fenugreek transcriptome

The raw RNA-sequencing data were obtained from the National Center for Biotechnology Information (NCBI) database with accession number PRJNA508420, comprising fenugreek plant samples treated with methyl jasmonate harvested after 6, 12, 24, 48, 72, and 120 h of treatment together with the control treatment [13] (Supplementary Table 1). All the sequencing reads passed the quality control step (Supplementary Fig. 1). Since there was no reference transcript for fenugreek plants, we pursued the *de novo* assembly approach. We assembled each sample independently using the Trinity software [15], and deposited the transcripts in the public repository of Zenodo. For the rest of the analysis, we used the assembled transcripts based on the RNA-seq data of the control sample since it has the highest BUSCO completeness score (Supplementary Tables 1&2). Thus, we mapped the sequencing reads of other samples on this assembled transcript set (see Method section for details).

### Differentially expressed genes of bioinformatics analysis show the significance of methyl jasmonate treatment in fenugreek

**Fig. 2:**
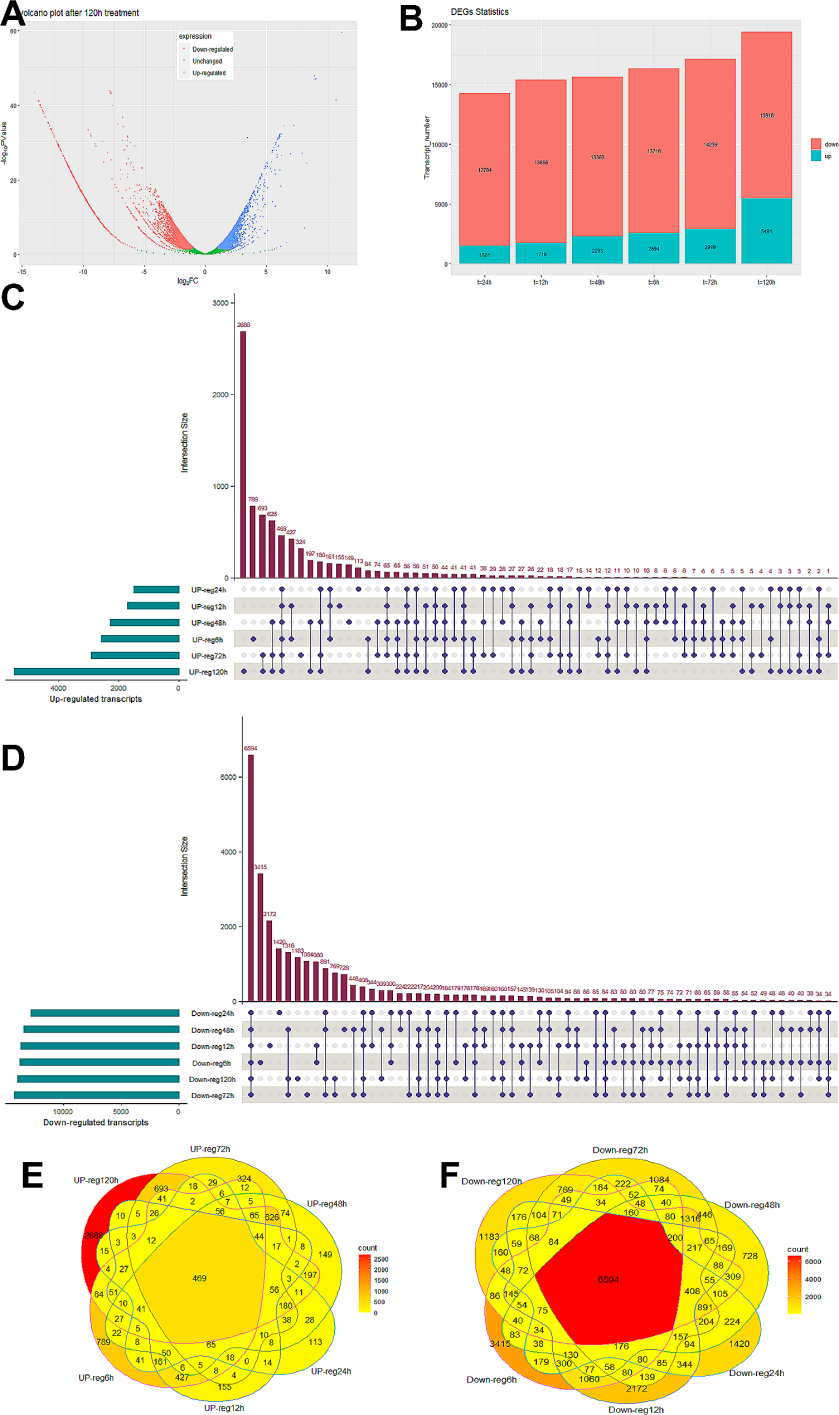
Bioinformatics analysis of assembled transcripts. (**A**). Volcano plot for up- and down-regulated transcripts after 120 h treatment with methyl jasmonate. Data points in green (close to zero) represent unchanged transcription level of a specific transcript compared to the control treatment. The blue points and red points represent the up- and down-regulated transcripts within that specific time point, respectively. (**B**). The stacked bar chart represents the number of up-regulated (cyan) and down-regulated (red) transcripts expressed after 6, 12, 24, 48, 72, and 120 h of methyl jasmonate treatment. (**C**). Intersection of up-regulated transcripts. (**D**). Intersection of down-regulated transcripts. (**E**). Distribution of transcripts with increased expression 6 (UP-reg6h), 12 (UP-reg12h), 24 (UP-reg24h), 48 (UP-reg48h), 72 (UP-reg72h) and 120 h (UP-reg120h) after methyl jasmonate treatment illustrated with a Venn diagram. (**F**). Distribution of transcripts with decreased expression

We analyze the expression profile of samples of six time points, namely, 6, 12, 24, 48, 72, and 120 h after methyl jasmonate treatment. To demonstrate the changes in the expression level of transcripts regulated by the methyl jasmonate application, we calculated the fold change (FC) in gene expression levels of different time points compared to the expression levels of the control sample (Fig. 2A and Supplementary Fig. 2). The volcano plot for the sample harvested after 120 h of treatment (Fig. 2A) shows the distribution of transcripts with increased or decreased expression levels. One can see that after 120 h the effect of methyl jasmonate on the plant is reduced resulting in a decrease in the fold change of expression (Supplementary Fig. 2). Besides, there are a few transcripts that experienced increased expression much more than the rest in the same timespan located on the top right side of the Volcano plot (Fig. 2A; Table 1). Of note, after 12 h of treatment, there are 2 transcripts with extreme increase in terms of expression level according to Supplementary Fig. 2 and Table 1. One of the transcripts is associated with the PAA1 protein belonging to the ATPase family, a group of enzymes that catalyze the breakdown of ATP to ADP or the reverse reaction [43].

**Table 1:** Transcripts with the highest increase in expression after the application of methyl jasmonate treatment associated to the point on the top right side of the Volcano diagram in Fig. 2A (FC: fold change)

Time Points	Transcript Name	logFC	P-value	Protein	Gene	Expression change
6 h	TRINITY_DN20315_c0_g2_i1	11.43	9.1e-63	-		Up
	TRINITY_DN15325_c0_g1_i1	12.13	4.2e-53	-		Up
12 h	TRINITY_DN20315_c0_g2_i1	11.25	7.9e-61	Copper-transporting ATPase PAA1, chloroplastic	PAA1	Up
	TRINITY_DN15325_c0_g1_i1	11.95	2.9e-51	Hydroquinone glucosyltransferase	AS	Up
24 h	TRINITY_DN20315_c0_g2_i1	11.89	4.7e-66	Auxin response factor 3	ARF3	Up
48 h	TRINITY_DN20315_c0_g2_i1	11.38	3.2e-62	Dynein light chain, cytoplasmic	DLCB	Up
72 h	TRINITY_DN20315_c0_g2_i1	10.33	2.1e-54	Spermatogenesis-associated protein 20	SPATA20	Up
120 h	TRINITY_DN20315_c0_g2_i1	11.15	2.9e-60	-		Up
	TRINITY_DN15325_c0_g1_i1	10.701	5.01e-42	-		Up

To better understand the functional aspect of methyl jasmonate treatments, we analyzed GO terms based on three categories including molecular functions, biological processes, and cellular components [16–17]. The number of transcripts that are involved in a specific function is shown in Table 2 (Full list in Supplementary Fig. 3). Of note, GO functions including metal ion binding, protein phosphorylation, proteolysis, and translation were found to be associated with the highest number of transcripts.

**Table 2:** The number of transcripts involved in each function of GO term in three categories in different time points

GO term Classification	GO terms	Control	6 h	12 h	24 h	48 h	72 h	120 h
Molecular function	Protein binding	3831	1934	3187	-	-	3912	3696
	ATP binding	3524	1828	2945	2963	3034	3643	-
	Protein kinase activity	1791	892	1583	1617	1615	1943	1914
	DNA binding	1231	632	1115	1138	1082	1361	1265
	RNA binding	-	593	817	802	803	1067	888
	Zinc ion binding	935	381	714	781	774	982	844
	Nucleic acid binding	793	328	626	688	683	904	800
	Oxidoreductase activity	678	479	579	629	593	746	663
	Metal ion binding	767	378	568	627	619	734	668
	Catalytic activity	416	324	441	486	470	607	536
Cellular components	Membrane	1463	771	1210	1348	1262	1504	1485
	Integral component of membrane	1204	674	1008	1055	978	1267	1144
	Nucleus	314	175	309	296	279	368	335
Biological processes	Protein phosphorylation	1795	896	1593	1627	1630	1952	1925
	Trans-membrane transport	1112	548	930	1032	950	1157	1137
	Regulation of transcription	1092	574	969	971	907	1111	1075
	Carbohydrate metabolic process	656	394	502	513	508	658	614
	Proteolysis	536	267	398	434	447	495	522
	translation	656	261	292	299	268	348	300

### Several of up- or down-expressed transcripts are in common at different time points after treatment

We calculated the number of up- and down-regulated genes in each time point sample compared to the control (Fig. 2B). The highest number of transcripts with increased expression occurred for the sample of 120 h after treatment with methyl jasmonate (i.e., 5491 transcripts, which is 2.1 higher than the number of up-regulated transcripts at 6 h after treatment). The highest number of down-regulated transcripts corresponds to the sample at 72 h after treatment with 14,239 transcripts. Besides, in the 24 h methyl jasmonate treated plants, the lowest number of transcripts were upregulated (i.e., 1501 transcripts). Cumulatively, the highest number of transcripts with modified expression levels (regardless of being up- or down-regulated) was in 120 h (19,409 transcripts), while the lowest in 24 h (Fig. 2B). Besides, Fig. 2C(D) shows the extent of shared transcript among different time points that were up (down) regulated with 469 (6594) genes in common indicating a high variation in the number of transcripts. Venn diagrams in Fig. 2E-F show the numbers of transcripts in common between different time points with increased and decreased expression, respectively. We can see that 469 (6549) transcripts with increased (decreased) expression levels were in common at all time points (center of Fig. 2E&F), showing the diversity of changes in expression levels.

### Transcription level of genes involved in the diosgenin biosynthesis is affected by methyl jasmonate treatment

**Fig. 3:**
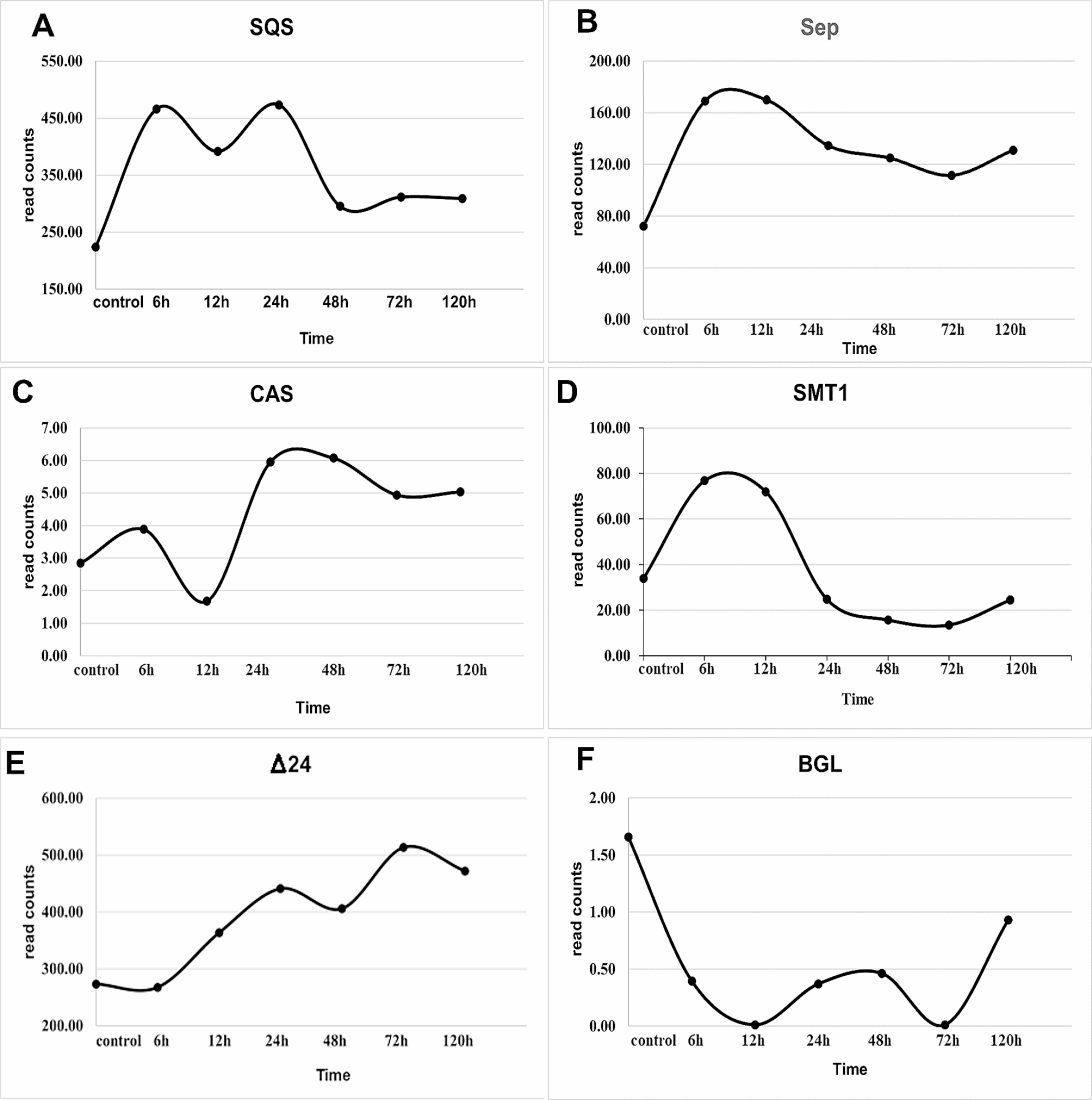
Dynamics of expression for six genes after the treatment. (**A**) Squalene synthase (SQS), (**B**) Squalene Epoxidase (SEP), (**C**) Cycloartenol Synthase (CAS), (**D**) Sterol Methyltransferase 1 (SMT1), (**E**) Delta24-sterol reductase (Δ24), (**F**) Beta-glucosidase (BGL)

Among all transcripts assembled, we identify candidate genes involved in the diosgenin biosynthesis [18] including SQS, SEP, CAS, SMT1, Δ24, and BGL (Fig. 1). Our results showed an increase of transcription level over time for most of the candidate genes. However, the only gene that showed a decreasing pattern over time was BGL (Fig. 3F). Besides, Fig. 3D shows an increase in the transcription level of SMT1 followed by a decreasing pattern after 6 h. The lower levels of BGL might be due to the fact the plant might prefer to accumulate the glycosylated form of diosgenin rather than its free form. It has been also previously shown that fenugreek plants are able to accumulate diosgenin in its glycosylated form; in the biosynthesis pathway of diosgenin, Glycoside saponins such as dioscin convert to diosgenin during the hydrolysis process [19].

Squalene synthase (SQS) is present in the first step of the biosynthesis pathway of diosgenin (Fig. 1). After applying methyl jasmonate, an increase in its expression can be seen with some fluctuations (Fig. 3 and Supplementary Fig. 4). At 6 h, its expression is two times higher than that of the control sample. The final decrease might be due to the fact that SQS is placed upstream of the whole pathway and the effect of the applied elicitor gradually diminished. Next, Squalene Epoxidase (SEP) oxygenates the product of the previous step, i.e., squalene. The application of methyl jasmonate elicitor increases the intermediate compound 2,3oxidosqualene metabolite by affecting the squalene metabolite. SEP expression witnessed an increase first, followed by a slight decrease staying at a level higher than the control (Fig. 3).

Cycloartenol Synthase (CAS) converts 2,3oxidosqualene into cycloartenol. Its expression is first reduced and then increased at the later stages of the methyl jasmonate application (Fig. 3). It is implied that some intermediate pathway genes are affected later than the ones at the beginning of the pathway and hence are influenced later. Often the production of the previous compound in the pathway (in this case, 2,3oxidosqualene) might act as an enhancer of the CAS expression levels which needs time within such a multistep pathway. This gene is known as a rate-limiting enzyme, a key enzyme whose activity determines the overall rate of the metabolic pathway. A decrease in its expression probably causes methyl jasmonate to initially increase with having little effect on CAS, but then, methyl jasmonate can affect the CAS from upstream pathways justifying the later increase. Another important gene is sterol Methyltransferase 1 (SMT1) located in the pathway of diosgenin biosynthesis after CAS. This gene produces 24-methylene cycloartanol in a parallel pathway with Δ24 reductase. In 6 h after the treatment, there was an increase in SMT1 gene, followed by a decrease, reaching a lower level than the control.

After the effect of CAS on 2,3oxidosqualene and the production of cycloartenol metabolite, Δ24 facilitates cycloartanol formation (Fig. 1). RNA-seq data proved a continuous increase in Δ24 levels over time (Fig. 3). The increase in the expression levels of this gene reaches approximately 1.6 times the value at 72 h after the treatment. Next is Beta-glucosidase (BGL) which belongs to the glycosidase enzyme family found in many organisms. These are converted in various processes, including the metabolism of sugars, biomass, phytohormones activity, and cell wall ligninization with the involvement of plant chemical defense against pathogens [19]. Therefore, it is less likely to be present freely in the plant. Its expression is the lowest at 72 and 48 h after applying the treatment (Fig. 3).

### Validation of bioinformatics analysis using real-time PCR

To validate the obtained results from the bioinformatics analysis we have grown three accessions of fenugreek plants in the greenhouse with the same conditions described by Zhou [7]. For the real-time PCR reaction, we used 4 genes including SQS, SEP, CAS, and SSR (Δ24). The results of the real-time PCR analyses confirmed that the overall trend of the expression of these genes is increasing. However, we are aware of differences in culture conditions (e.g., altitude, relative humidity, and genetic background of accessions) of the current study with the one where RNA-seq analysis was performed expecting minor effects on the results.

#### Relative gene expression of Squalene synthase (SQS) and fold changes compared to the control

**Fig. 4:**
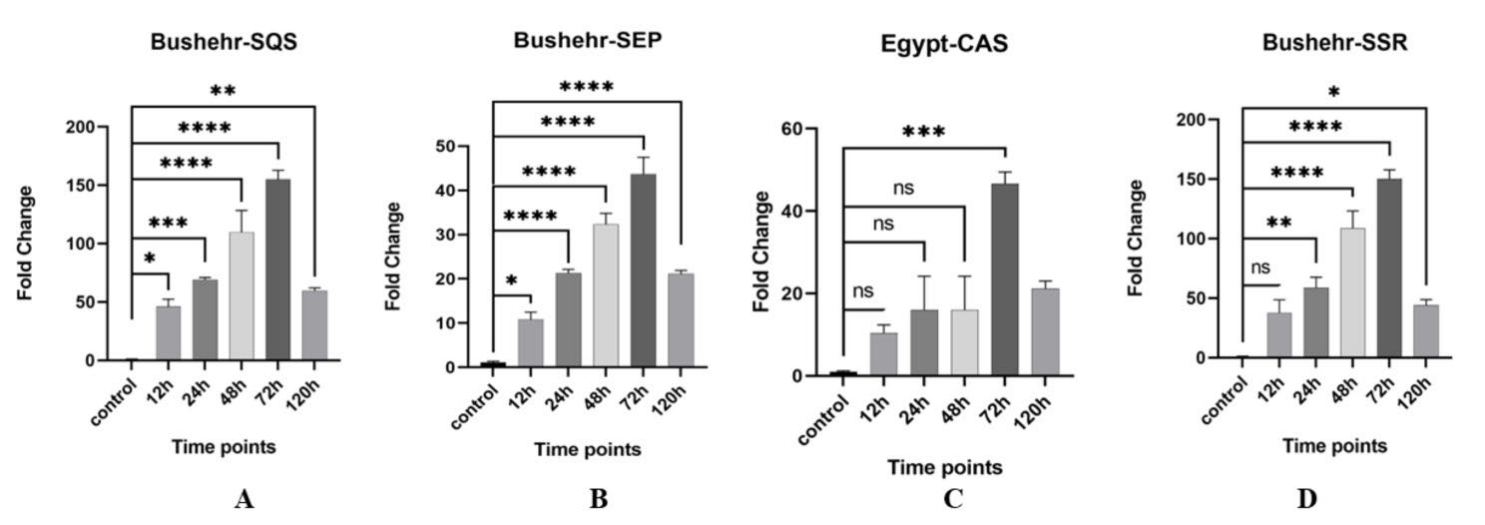
The expression profile of three populations after methyl jasmonate treatment (ns: not statistically significant, *: from 0.01 to 0.05, **: from 0.001 to 0.05, ***: from 0.0001 to 0.05, ****: less than 0.0001)

SQS gene synthesizes one molecule of squalene by connecting two molecules of farnesyl pyrophosphate, which is the first committed step in the biosynthesis of sterols. We studied the relative expression of SQS in three fenugreek populations of Shiraz (Iran), Bushehr (Iran) and Egypt at different time points after treatment with methyl jasmonate (Table 3; Fig. 4A). The highest fold change corresponds to Bushehr’s sample at 72 h after treatment (FC = 155.03) and the lowest was related to Shiraz’s sample at 12 h after treatment (FC = 0.803). The general trend is an increase compared to the control, except at the time of 120 h after treatment. This result is consistent with the results of bioinformatics analysis, which shows an increase in gene expression in 72 h after treatment.

**Table 3:** Fold changes of the SQS, SEP, CAS and SSR genes at different time points after methyl jasmonate treatment

Gene Name	population	12 h	24 h	48 h	72 h	120 h
SQS	Shiraz	1.39	3.73	2.34	1.80	0.80
	Bushehr	59.56	155.03	109.80	69.13	45.92
	Egypt	7.86	26.60	9.12	9.12	8.09
SEP	Shiraz	12.19	31.35	17.86	13.14	6.43
	Bushehr	21.16	43.67	32.36	21.31	10.79
	Egypt	9.71	15.59	8.18	8.18	5.42
CAS	Shiraz	14.63	33.51	24.93	17.43	10.90
	Bushehr	16.39	45.35	37.02	24.47	14.85
	Egypt	21.24	46.60	15.99	15.99	10.40
SSR	Shiraz	3.79	6.20	8.87	4.26	3.93
	Bushehr	37.62	58.75	108.98	150.09	44.07
	Egypt	3.47	5.24	5.24	15.36	4.76

#### Relative gene expression of SEP, CAS and SSR

SEP gene performs the first oxygenation step in the biosynthesis of sterols by epoxidizing the squalene molecule. Because this gene is upstream of the diosgenin biosynthesis pathway, it can be one of the effective genes in increasing diosgenin metabolite. The results of the minimum significant difference analysis show a significant difference in the Bushehr mass at 72 h after applying the treatment compared to the control (at the significance level of 0.05). The highest multiplication changes occurred in the Bushehr mass at 72 h after treatment, 45 times higher than that of the control. The highest fold changes compared to the control of SEP is for the sample of Bushehr at 72 h after treatment (FC = 43.67) reported in Table 3; Fig. 4B. However, the lowest changes related to the sample Egypt at 12 h after the application of treatment (FC = 5.42).

CAS converts 2,3-oxido-squalane to cycloartenol, which is a necessary substrate to convert to phytosterols and other plant sterols. The mass of Bushehr and Egypt at 72 h after applying the treatment has increased about 45 times compared to the control. In Bushehr and Shiraz, all time intervals except 12 h, have a significant difference with the control at the significant level of 0.05 (Table 3; Fig. 4C). The highest (lowest) fold change was seen for the Egypt sample 72 (12) hours after treatment.

The Δ24-reductase gene (*a.k.a.*, SSR) encodes the side branch of reductase by sterol enzyme and is responsible for converting cycloartenol to cycloartenol. It is considered the enzyme that determines the path of cholesterol synthesis [20]. Bushehr population has a significant difference with the control (at the significant level of 0.05) in all intervals except 12 h after treatment. The highest fold changes occurred in the Bushehr sample at 72 h after treatment (about 150 times) compared to the control. However, the lowest fold changes compared to the control was 3.47 for the sample of Egypt 12 h after treatment (Table 3; Fig. 4D). The general trend of fold changes for all three genes is an increase, except for 120 h after the treatment (compared to the control).

## Discussion and conclusion

Fenugreek is known as a medicinal plant and a source of a biogenic steroid which is important in the pharmaceutical industry. The two main secondary metabolites in fenugreek are trigonelline and diosgenin. Diosgenin is a saponin found in fenugreek, in addition to its many therapeutic effects, known as the most active compound of fenugreek [21]. However, the amount of this metabolite in the fenugreek plant is low. Treatment with methyl jasmonate can result in increased expression of genes involved in the diosgenin biosynthesis which subsequently is reflected in an increase in the amount of this substance. To investigate the effect of methyl jasmonate on the increase or decrease of diosgenin, we investigated its effect on the genes involved in the diosgenin biosynthesis. For this purpose, the RNA-seq datasets deposited in the NCBI database were used with the Trinity software to assemble the transcriptome.

Differential gene expression analysis revelaed a decrease in the BGL gene expression pattern over time after methyl jasmonate application. also performed. This trend is likely due to plants tendency to preserve diosgenin in its pure form by preventing the formation of glucose molecules. For this purpose, the BGL enzyme should be less active resulting in its expression reduction. The increased count of up- and down-regulated transcripts at the 120-hour may result from the influence of methyl jasmonate on additional biosynthetic pathways and biological processes. These pathways may either exhibit lower sensitivity to the treatment or rely on intermediate metabolites generated throughout the process. This aligns with our anticipated effects of methyl jasmonate on gene expression. Upregulation of SQS and SEP in response to methyl jasmonate has been describedin other plants like *Panax ginseng*. Additionally, the application of methyl jasmonate is known to elevate diosgenin levels in *T. foenum-graecum* [22]. To validate the accuracy of the bioinformatics analysis, real-time PCR was conducted in the laboratory. As expected, an increase in SQS and SEP gene expression was observed. In the Bushehr population, both genes of SQS and SEP showed significant elevation 72 h after treatment ( expressed 155 and 43 times higher than the control sample) confirming the bioinformatic findings. The Egypt population showed the highest fold changes for the CAS gene expression at the same time point, consistent with previouslyy observed bioinformatic trends. Notably, the real-time PCR analysis showed a substantial150-fold increase in the SSR key gene, aligning with the bioinformatics results. Since this is a key gene in the diosgenin biosynthesis pathway, increasing its expression can have a direct effect in producing more diosgenin as the final product of the pathway.

Two pivotal genes, Δ24 and SMT1, which take part in the branch point, exhibited relatively contrasting transcriptional expression patterns. The gene expression result, alongside with diosgenin content, suggested that the Δ24 plays an essential role in directing the metabolic flux toward diosgenin production, further validating our findings. Conversely, our results clearly indicates the opposite impact of SMT1 in the diosgenin biosynthesis pathway. Previous studies on transgenic plants demonstrated that the over-expression of SMT1 results in decreased cholesterol production, while its down‐regulation causes higher biosynthesis of cholesterol.Real-time PCR results also revealed that a high expression of Δ24 resulted in a higher amount of diosgenin, whereas increased SMT1 expression is associated with reduced diosgenin production. This suggests that the branch point regulates diosgenin biosynthesis [1].. The obtained results show that SMT1 had a significant downward trend in expression after 12 h with minimum increases at120 hours, while Δ24 expression gene exhibited an increasing trend. Overall, our results indicate that the elicitor positively regulates the diosgenin biosynthetic pathway genes, particularly SMT1 and Δ24, potentially enhancing the final diosgenin yield in fenugreek plants.

Overall, the bioinformatic data were validated by real-time PCR experiments, indicating the precision of the RNA-sequencing data analysis. This valuable agreement indicates the accuracy of the RNA-seq analyses. It might not be always the case to have complete agreement between *in silico* analysis and wet lab results for all samples and all time points since the populations, cultivation, and greenhouse conditions and several other factors can affect the results. However, the general trend of gene expression in this study for the investigated genes was in agreement using both approaches.

## Materials and methods

### RNA sequencing data for fenugreek

In this study, RNA sequencing data for fenugreek were downloaded from the SRA of the NCBI database. In addition to the control sample of the fenugreek plant with accession code SRR8281656, there are six time points after treatment with methyl jasmonate including 6 h (SRR8281657), 12 h (SRR8281658), 24 h (SRR8281659), 48 h (SRR8281660), 72 h (SRR8281654) and 120 h (SRR8281655) which all come from high throughput sequencing machine, Illumina HiSeq 2000. All the reads are paired end with a length of 150 bp each. The SRA toolkit v3.0 was used to download the raw sequencing reads in SRA format with command line *prefetch* in the Linux operating system. This is followed by fastq-dump to convert them to FASTQ format. Then, FastQC v0.11.8 was used to check the quality of the downloaded sequencing reads.

### The de novo transcriptome assembly

The Trinity software package v2.15 [15] was used to assemble the raw sequencing reads of fenugreek [13]. This took 73 CPU hours using 64GB of RAM. The *TrinityStats.pl* script from the Trinity package was used to report the assembly statistics including median contig length, GC percentage, contig N50, and total assembled bases. To evaluate the completeness of the assembled transcripts, BUSCO v4.12 (with argument -m transcriptome) was used with the eukaryota_odb10 dataset. We looked for the transcript level variation of the selected genes. For this purpose, we calculated FPKM (fragments per kilobase exon per million mapped fragments) at different time points after methyl jasmonate treatment.

### BlAST analysis and GO enrichment

To compare assembled transcripts with proteins that are available in public databases, Basic Local Alignment Search Tool (BLAST) v2.14 was used against the universal protein resource knowledgebase (UniProtKB) including around 249 million sequences across the Tree of Life. In order to analyze the transcriptomes in terms of functional annotation, the Trinotate software v4.0 [23] was used, and Gene Ontology (GO) [16–17] terms associated with the assembled transcripts were found.

### Differential expression gene analysis

The raw sequencing reads of all samples were mapped to the assembled transcripts of the control samples using the bowtie2 software v1.3. Then, using the samtools package v.1.12, mapped sequences were extracted and compressed to produce the BAM file. Then, the Express package v1.5 was used to quantify the number of reads that are mapped to the transcripts resulting in the expression profile. Statistical analysis and visualization were done using R software v4.1 and Rstudio v1.4 based on in-house scripts. To estimate DEGs, the deseq2 package v1.38 in R was used, resulting in the fold changes and p-values [24]. Results were visualized using the ggplot2 package v3.4, ggVennDiagram v1.2, and UpsetR v1.4. In a Volcano diagram, the most upregulated (or downregulated) genes are toward the right (or left). The p-value threshold was 0.05 and the log fold change of > 1 was considered as the expression change threshold. Since the p-value has a negative transformation, there is an inverse relationship between the y-axis and the p-value. Besides, each point corresponds to each transcript and when a point is close to the center, it indicates a gene with an unchanged expression level. Genes with statistically significant increased expression are shown in blue. However, down-regulated transcripts are plotted in red.

We also benefited from the UpsetR plot to show the number of transcripts that were in common among all time points or were present in only a few time intervals [25, 26]. Each point in a row of the UpsetR plot shows the number of genes with increased expression level at one time point (or a few time points), but without expression changes in the rest of the time points.

### Real-time PCR reaction

Real-time PCR reaction was performed in the laboratory to study the expression of genes including SQS, CAS, SEP, and SSR. Three plant populations including Shiraz (Iran), Bushehr (Iran), and Egypt were considered. Plants were treated with methyl jasmonate at a concentration of 0.015% and in time intervals of 12, 24, 48, 72, and 120 h after the treatment. They were removed and placed in a -80 °C freezer with liquid nitrogen. Then RNA was extracted from plant samples and the extracted RNA was converted into cDNA. Then, real-time PCR reaction was performed. First, it started with a 15-minute reaction at 95 °C followed by 35 cycles of 20 s at 95 °C and 35 cycles at 60 °C. Data were analyzed using the Livak analysis method (RQ2 = 2^-ΔΔCT^) [27]. The gene glyceraldehyde 3-phosphate dehydrogenase (GAP) was used as the reference gene [12, 14]. Because the number of samples was small, the Shapiro-Wilk normality test was used [8]. One-way ANOVA and non-parametric tests were used in the Prism software for variance analysis to compare the differences among groups of data. The results are reported as mean ± SE (standard error).

### Electronic supplementary material

Below is the link to the electronic supplementary material.

Supplementary Material 1

## Data Availability

The datasets analyzed during the current study are available in the Zenodo open repository at 10.5281/zenodo.8155183 and the NCBI database at https://www.ncbi.nlm.nih.gov/bioproject/PRJNA508420.
