# Supplementary material for: Transcriptomic data reveals the dynamics of terpenoids biosynthetic pathway of fenugreek
Source: BMC Genomics. 2024 Apr 22;25:390. doi: 10.1186/s12864-024-10253-x (PMC11034138; doi:10.1186/s12864-024-10253-x)
Supplement: Supplementary file 1 — Supplementary Material 1 [file 12864_2024_10253_MOESM1_ESM.docx]

**Transcriptomic data reveals the dynamics of terpenoids biosynthetic pathway of fenugreek**

In this section, there is a summary for the quality control checks of the bases (**Supplementary Figure 1**). And volcano plots for other time points after treatment with methyl jasmonate **(Supplementary Figure 2)**, Also We put the figures of the GO terms for the rest of time points after applying the treatment (**Supplementary Figure 3**). And there is a Heatmap at the end of the section (**Supplementary Figure 4**).

| SRR8281654 | 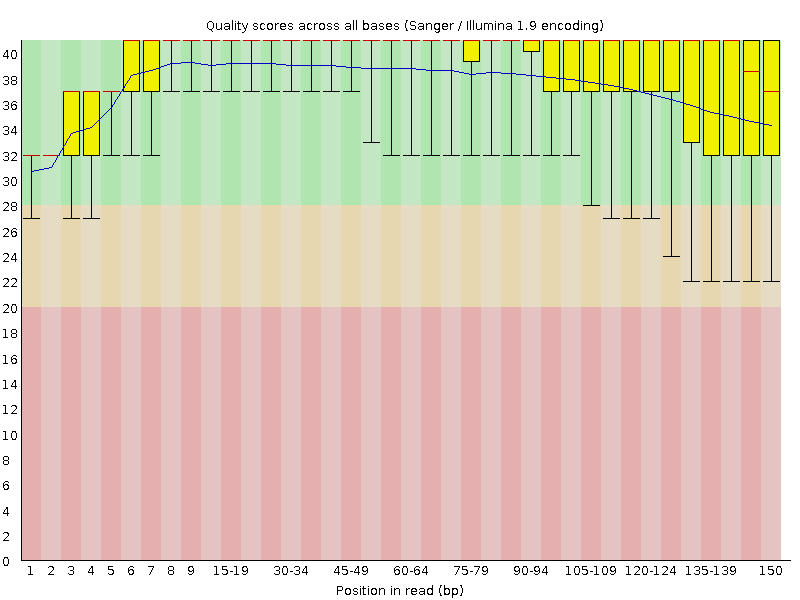 | 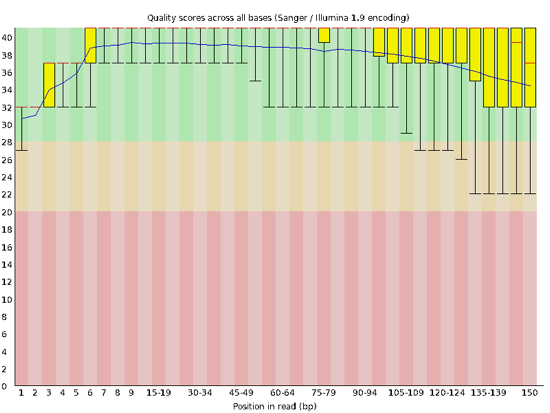 |
| --- | --- | --- |
| SRR8281655 | 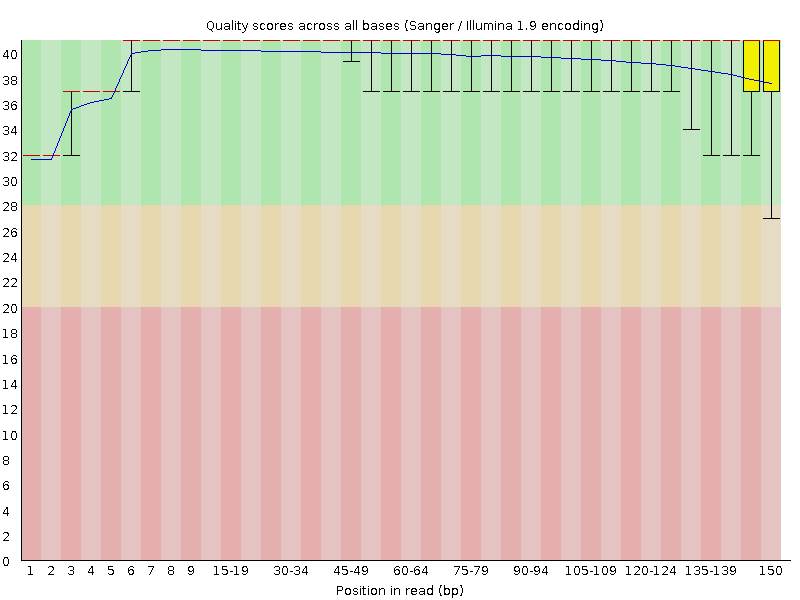 | 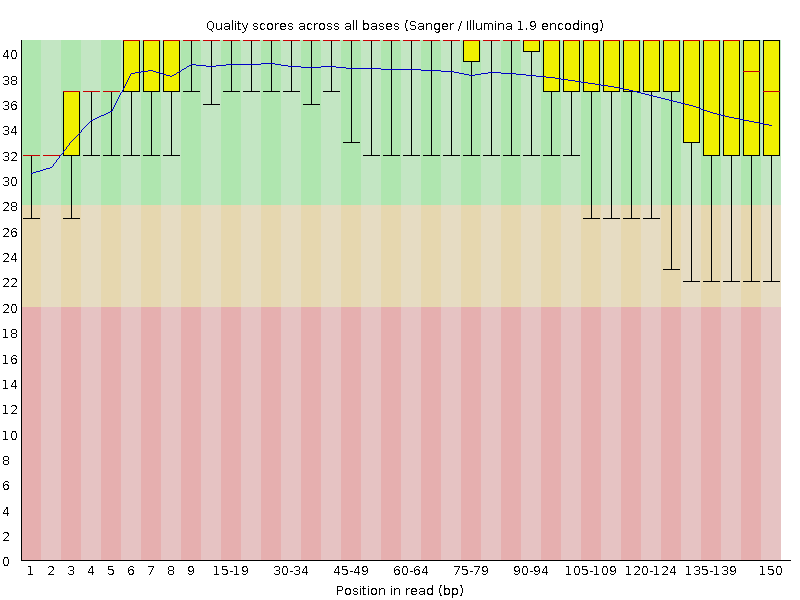 |
| SRR8281656 | 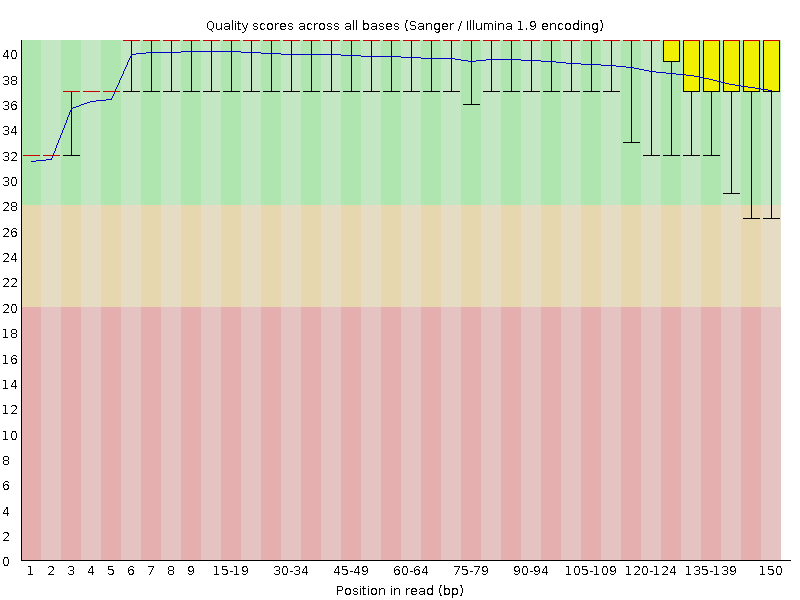 | 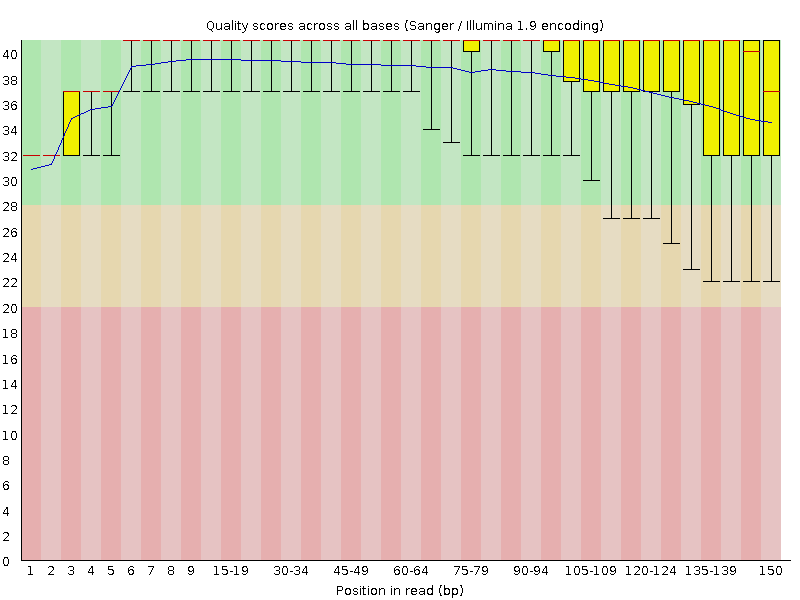 |
| SRR8281657 | 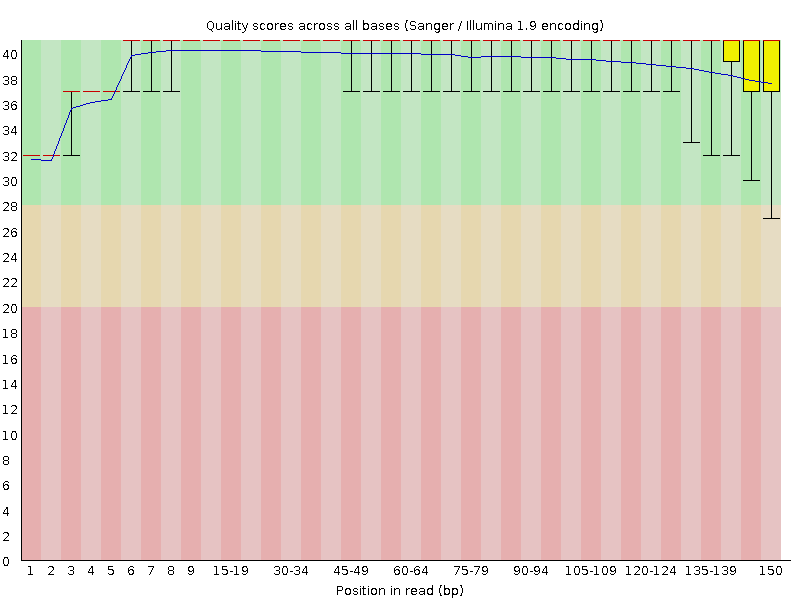 | 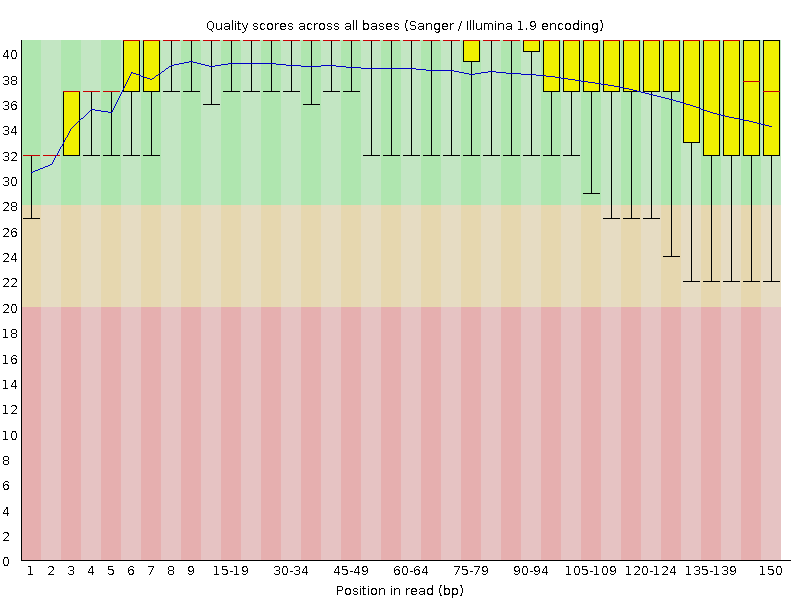 |

| SRR8281658 | 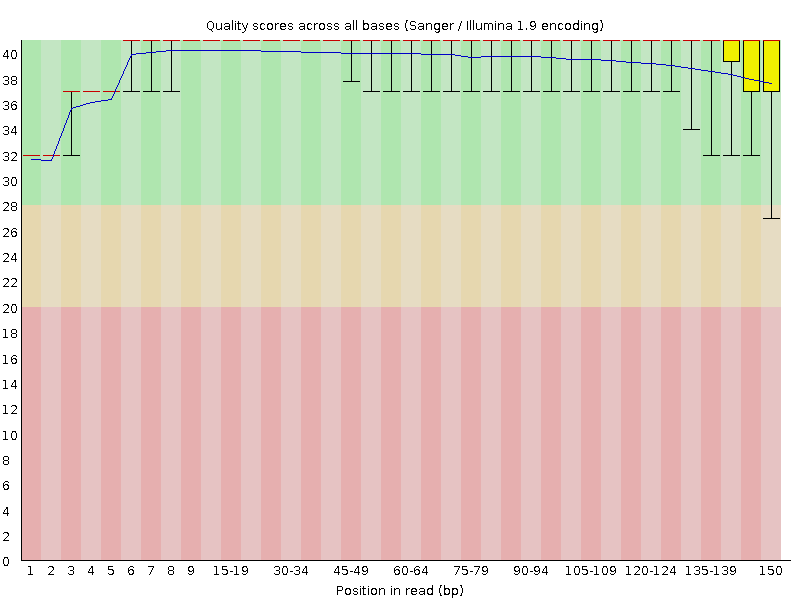 | 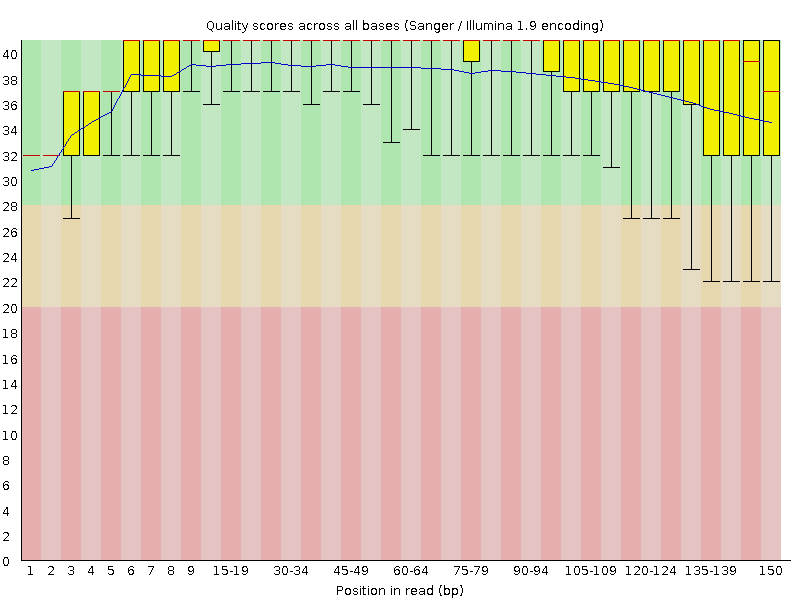 |
| --- | --- | --- |
| SRR8281659 | 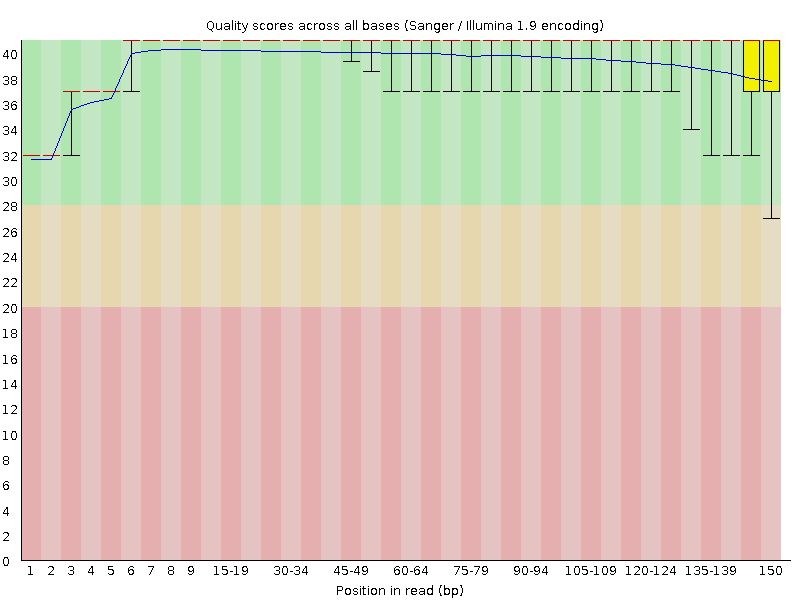 | 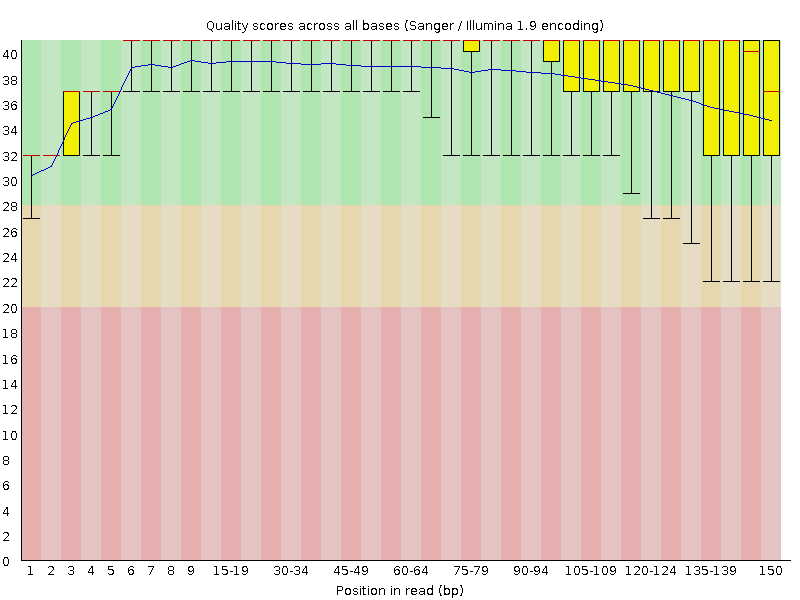 |
| SRR8281660 | 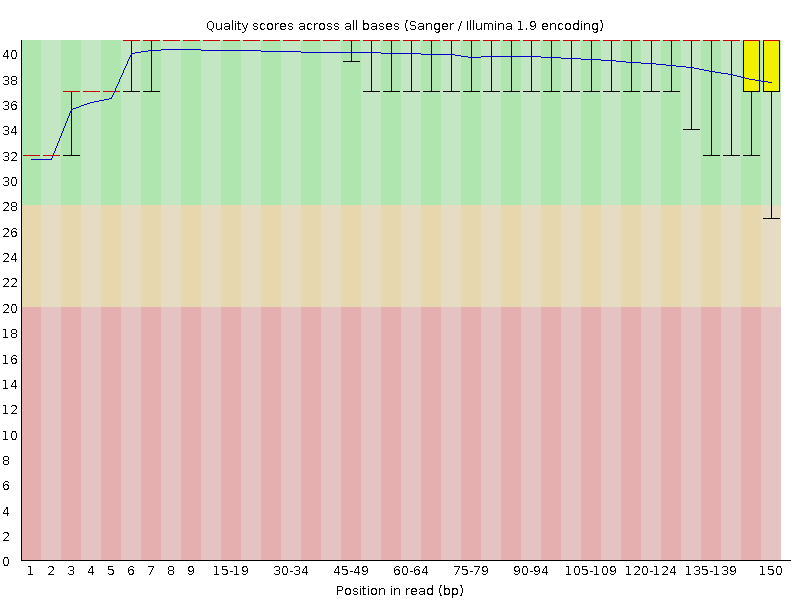 | 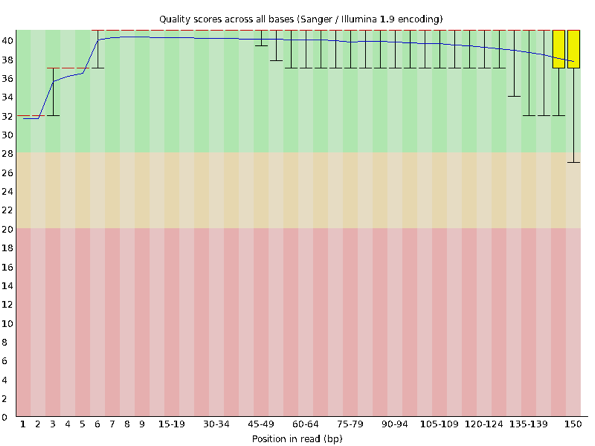 |

**Supplementary Figure 1:** Quality control of Sequencing reads for control sample and samples harvested after 6, 12, 24, 48, 72 and 120 hours of treatment. All reads are with high quality of Q20, which means that error is less than 1%.


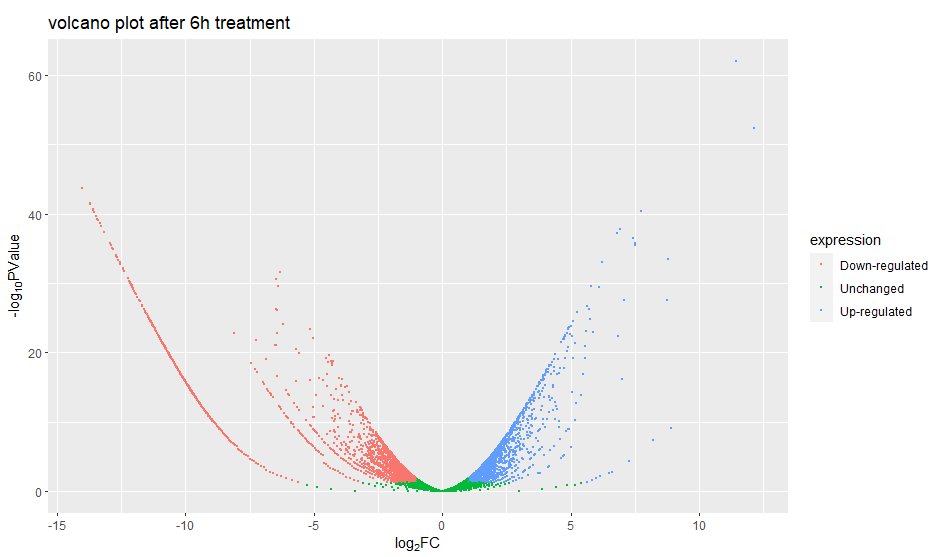

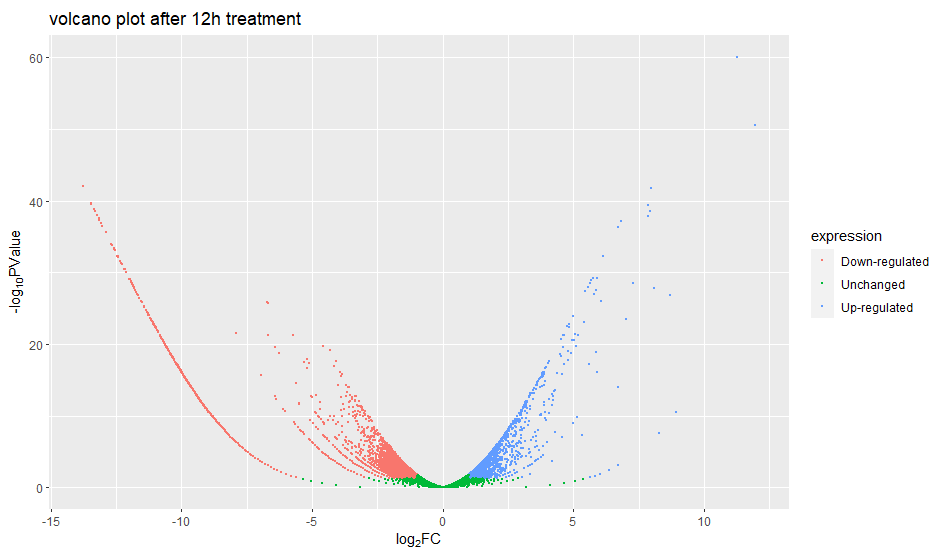


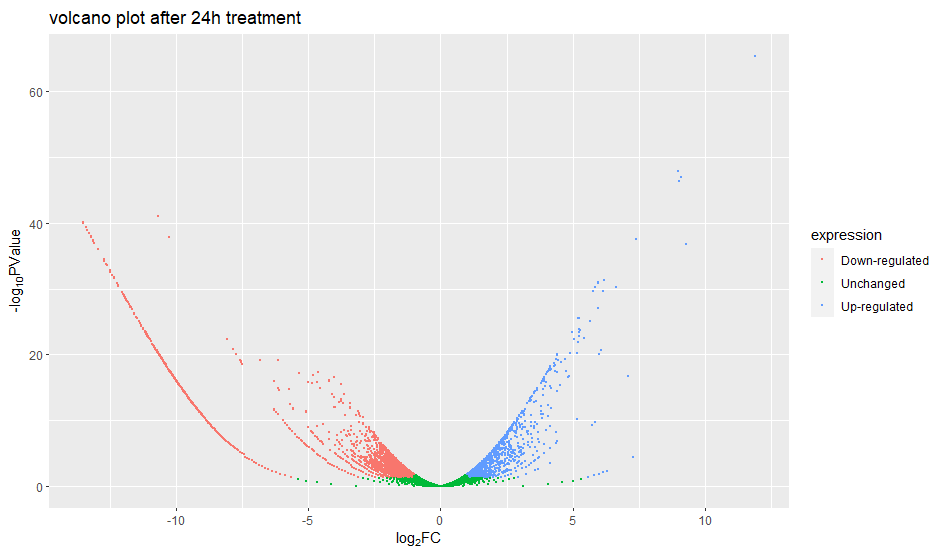

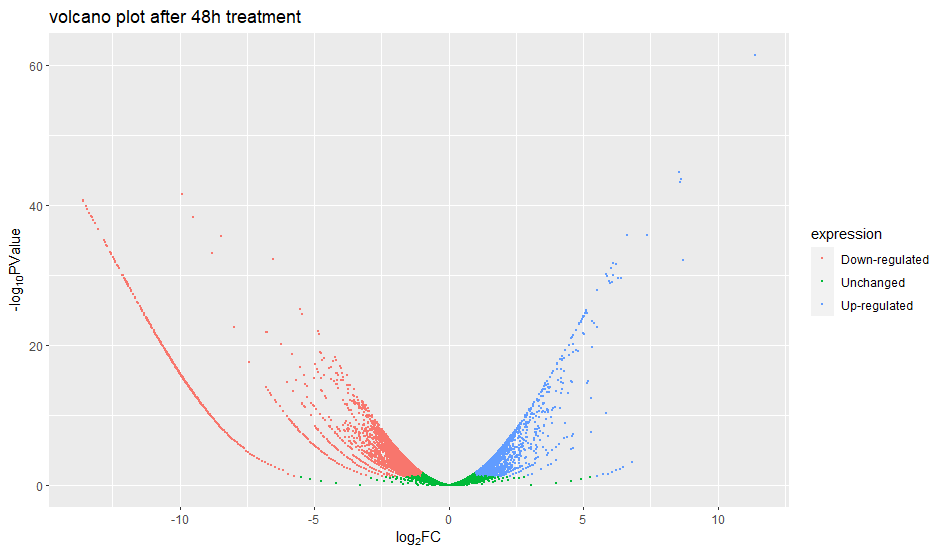


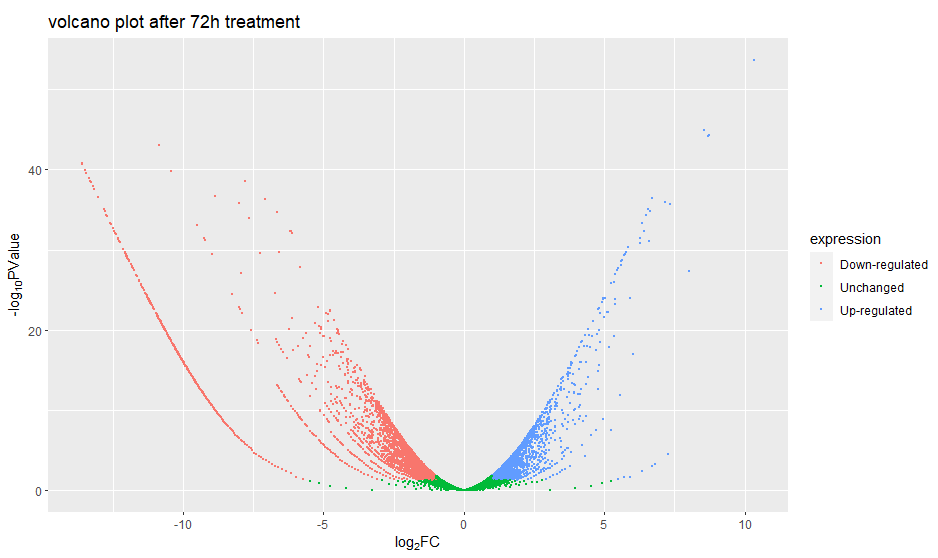


**Supplementary Figure 2.** Volcano plots for all time points.


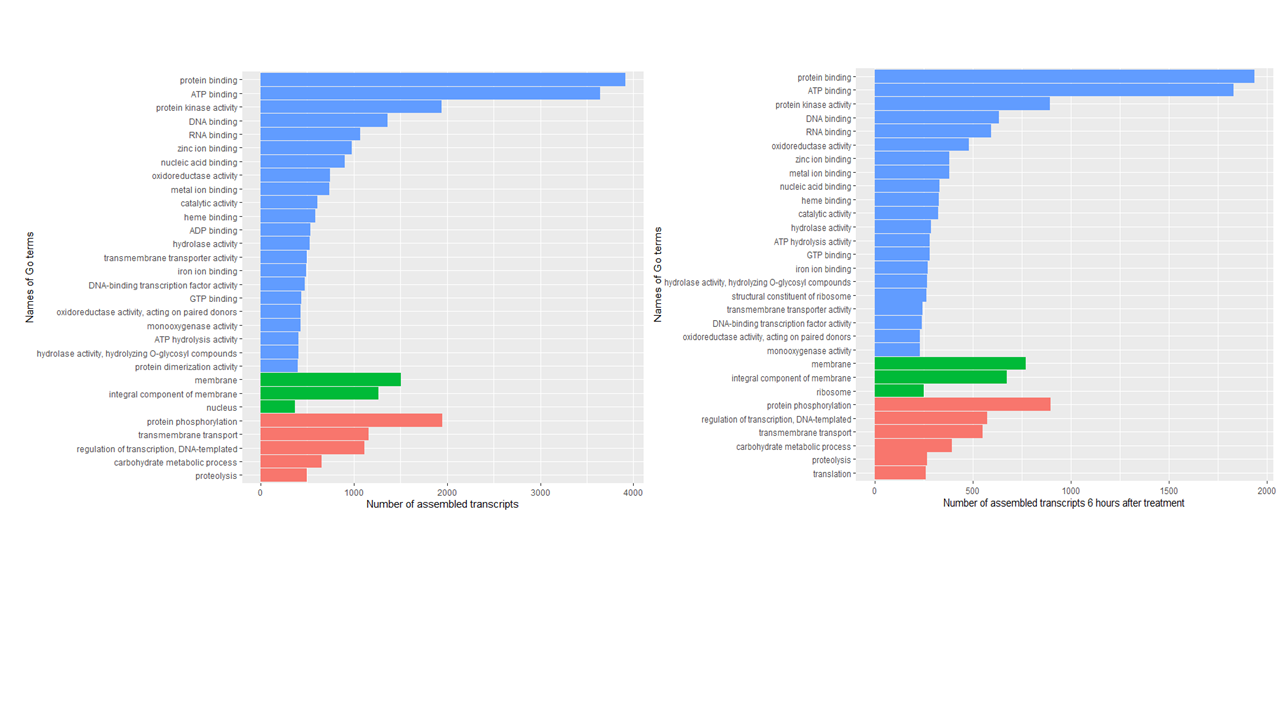


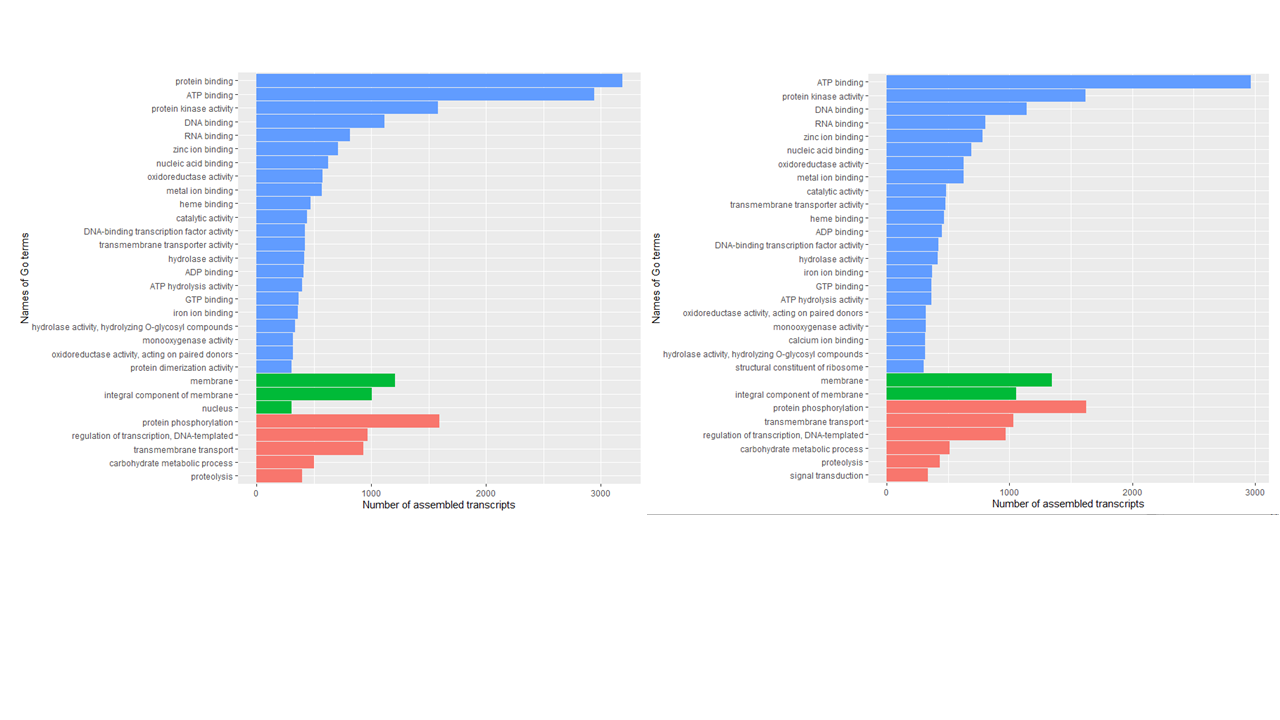

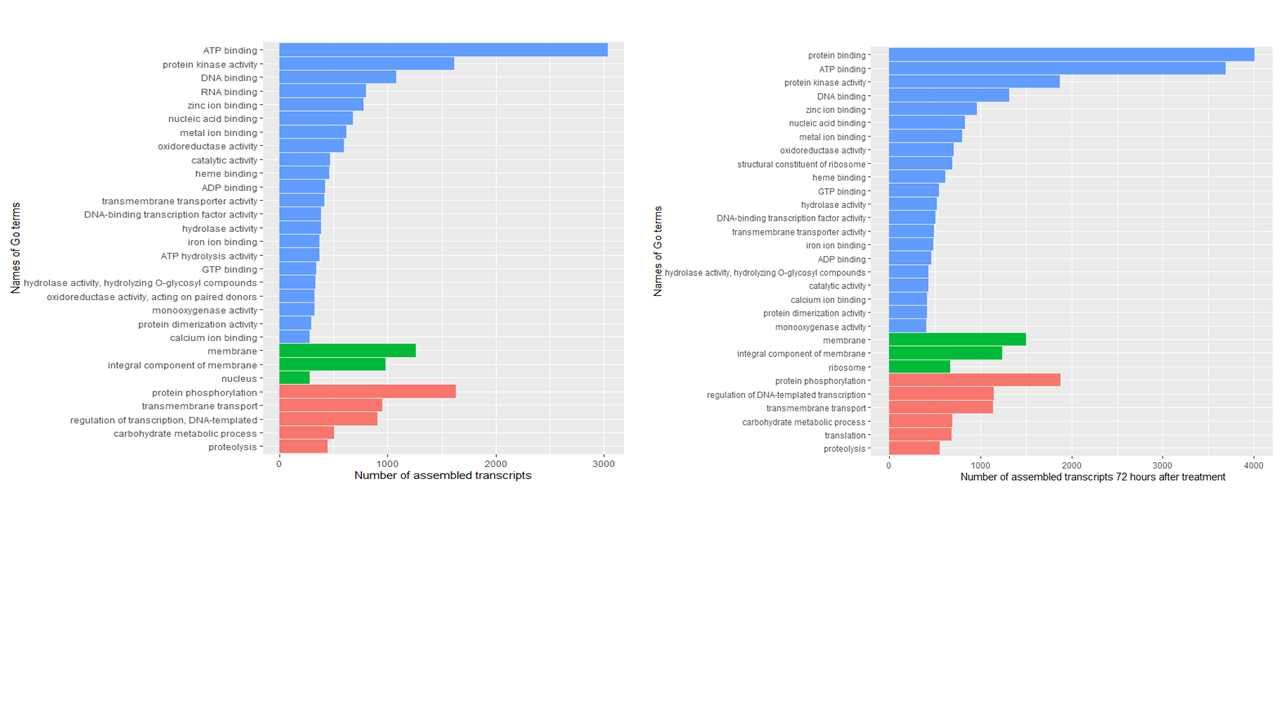

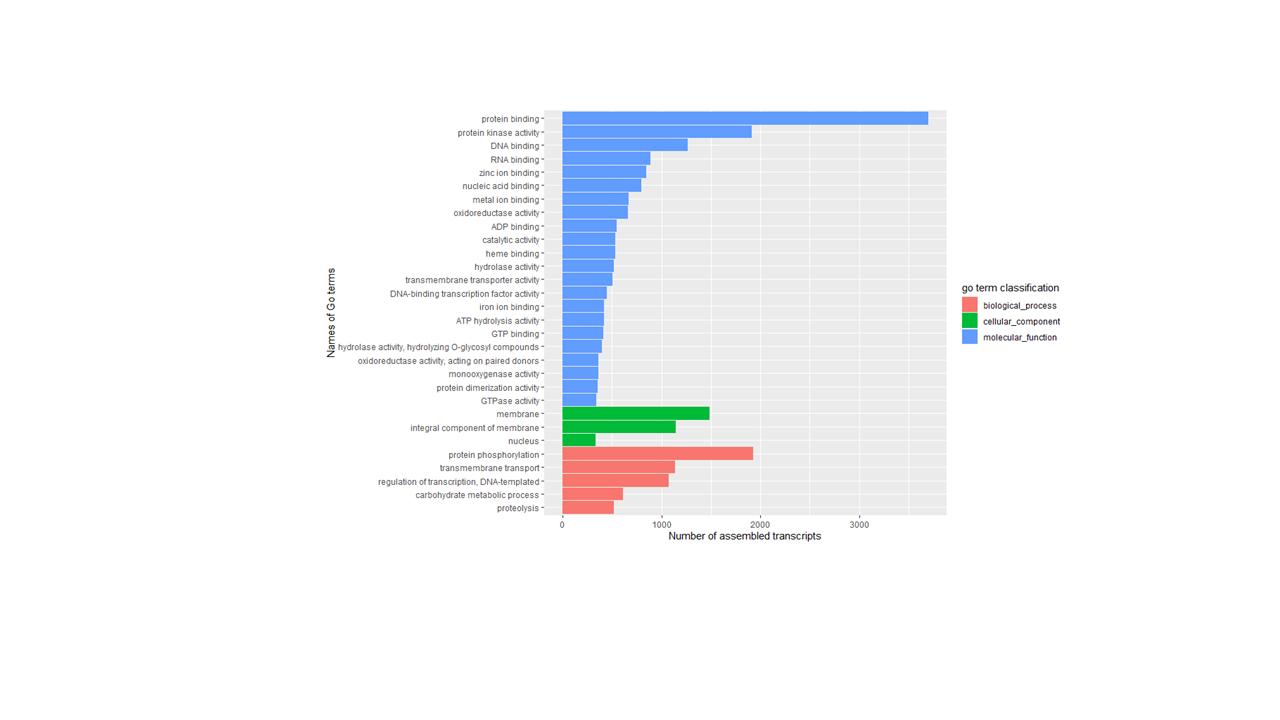


**Supplementary Figure 3.** Comprehensive list of GO terms.

In the GO term diagram related to 6 hours after treatment, in the category of GO-term molecular functions related to protein binding, the highest number of transcripts was about 1900. Also, in the category of biological processes, the highest number of transcripts was related to protein phosphorylation and less than half of the control chart, i.e., 800 transcripts. And in the GO term diagram related to 12 hours after treatment, in the category of molecular functions of GO-term related to protein binding, the highest number of transcripts was about 3200. Also, in the category of biological processes, the highest number of transcripts is related to protein phosphorylation, and it is twice the number of 6 hours chart, i.e., 1600 transcripts. In the graph of Go term related to 24 hours after applying the treatment, in the category of molecular functions of Go term related to ATP binding, the highest number of transcripts was about 2900. Also, in the category of biological processes, the highest number of transcripts was related to protein phosphorylation with about 1700 transcripts and almost similar to the 12-hour chart. In the GO term diagram related to 48 hours after applying the treatment, in the category of molecular functions of GOTERM related to ATP binding, the highest number of transcripts was around 3000 and almost the same as the 24 hours diagram. Also, in the category of biological processes, the highest number of transcripts was related to protein phosphorylation with about 1600 transcripts and similar to the 12-hour chart.

In the go term diagram related to 72 hours after treatment, in the category of molecular functions of go-term related to protein binding, the highest number of transcripts was 4000. Also, in the category of biological processes, the highest number of transcripts was related to protein phosphorylation with about 1800 transcripts. In the GO term diagram related to 120 hours after the application of the treatment, the highest number of transcripts was about 3700 in the category of GO term molecular functions related to protein binding. Also, in the category of biological processes, the highest number of transcripts was related to protein phosphorylation with about 1900 transcripts and similar to the control.


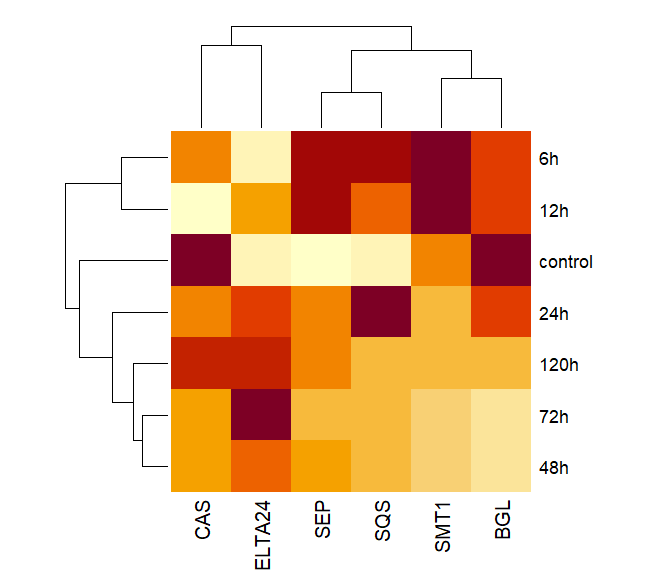


**Supplementary Figure 4.** The full heat map of genes in Figure 4

A heatmap is a graphical representation of data where values are depicted by color. It provides statistical tools for creating high-quality matrices**,** normalizing input data, running clustering algorithms**,** and visualizing results in dendrograms.

| **Supplementary Table 1.** *De novo* assembly description of SRAs from fenugreek plants treated with methyl jasmonate using Trinity. The total number of assembled transcripts obtained for the control sample is 114,414 covering more than 110 million bases. However, these values are 144,619 and 118 million for the sample at 120h after treatment, respectively. | | | | | | | |
| --- | --- | --- | --- | --- | --- | --- | --- |
| **Assembly descriptions** | **Raw Read SRA code** | **total number of genes** | **average contig length** | **number of assembled bases** | **number of transcripts** | **N50** | **GC Percent** |
| **Control** | [SRR8281656](https://trace.ncbi.nlm.nih.gov/Traces/sra/?run=SRR8281656) | 82,980 | 970.02 | 110,984,281 | 114,414 | 1,632 | 38.80 |
| **6 hours** | [SRR8281657](https://trace.ncbi.nlm.nih.gov/Traces/sra/?run=SRR8281656) | 48,656 | 738,29 | 38,811,837 | 52,570 | 1,028 | 39.70 |
| **12 hours** | [SRR8281658](https://trace.ncbi.nlm.nih.gov/Traces/sra/?run=SRR8281656) | 98,324 | 783.84 | 90,776,077 | 115,809 | 1,199 | 38.52 |
| **24 hours** | [SRR8281659](https://trace.ncbi.nlm.nih.gov/Traces/sra/?run=SRR8281656) | 105,285 | 778,87 | 97,727,392 | 125,474 | 1,203 | 38.37 |
| **48 hours** | [SRR8281660](https://trace.ncbi.nlm.nih.gov/Traces/sra/?run=SRR8281656) | 115,194 | 795,10 | 111,062,860 | 139,685 | 1,244 | 38.29 |
| **72 hours** | [SRR8281654](https://trace.ncbi.nlm.nih.gov/Traces/sra/?run=SRR8281656) | 74,487 | 1066,93 | 120,604,455 | 113,039 | 1,788 | 38.46 |
| **120 hours** | SRR8281655 | 118,212 | 816.69 | 118,109,316 | 144,619 | 1,289 | 38,02 |

| **Supplementary Table 2**. BUSCO score in percentage for the assembled transcripts for fenugreek plants treated with methyl jasmonate We calculated the BUSCO (Benchmarking Universal Single-Copy Orthologs) scores to look for the presence or absence of highly conserved genes in an assembly. the number of genes inferred at the time point 120 hours after the treatment is 118,212 (Supplementary Table 1) and its completeness percentage is very high, 96.8%, but with very low fragmentation (2.7%) or missing genes (0.5%). The highest number of assembled bases is 120,604,455 bps related to 72 h after treatment. According to the BUSCO statistics, the most complete global single-copy orthologs after control correspond to 72 h after treatment with complete BUSCO is 98.4%, missing BUSCO 0.4%, and fragmented BUSCO 1.2%. | | | | | | | |
| --- | --- | --- | --- | --- | --- | --- | --- |
| **Time points** | **control** | **6 h** | **12h** | **24h** | **48h** | **72h** | **120h** |
| **Complete BUSCOs (C)** | 99.2 | 72.9 | 94.5 | 93.8 | 96.1 | 98.4 | 96.8 |
| **Complete and single-copy BUSCOs (S)** | 54.1 | 53.7 | 63.1 | 57.3 | 56.5 | 50.2 | 58.4 |
| **Complete and duplicated BUSCOs (D)** | 45.1 | 19.2 | 31.4 | 36.5 | 39.6 | 48.2 | 38.4 |
| **Fragmented BUSCOs (F)** | 0.8 | 20.0 | 4.3 | 4.3 | 3.1 | 1.2 | 2.7 |
| **Missing BUSCOs (M)** | 0 | 7.1 | 1.2 | 1.9 | 0.8 | 0.4 | 0.5 |
